# Impact of Xpert MTB/RIF on Outcomes of Adults Hospitalized With Spinal Tuberculosis: Findings From a Comparative Cohort in Beijing, China

**DOI:** 10.3389/fpubh.2022.901504

**Published:** 2022-06-17

**Authors:** Jun Fan, Jun An, Wei Shu, Kai Tang, Yuanyuan Shang, Yi Xue, Shibing Qin, Yu Pang

**Affiliations:** ^1^Orthopaedics Department, Beijing Chest Hospital, Capital Medical University/ Beijing Tuberculosis & Thoracic Tumor Research Institute, Beijing, China; ^2^Medical Records Department, Beijing Chest Hospital, Capital Medical University/ Beijing Tuberculosis & Thoracic Tumor Research Institute, Beijing, China; ^3^Clinical Center on TB Control, Beijing Chest Hospital, Capital Medical University/ Beijing Tuberculosis & Thoracic Tumor Research Institute, Beijing, China; ^4^Department of Bacteriology and Immunology, Beijing Key Laboratory on Drug-Resistant Tuberculosis Research, Beijing Chest Hospital, Capital Medical University/Beijing Tuberculosis & Thoracic Tumor Research Institute, Beijing, China

**Keywords:** spinal tuberculosis, outcomes, Xpert, adults hospitalize, China

## Abstract

**Background:**

Spinal tuberculosis (TB) is one of the most common forms of extrapulmonary tuberculosis, causing increased morbidity and lifelong disabilities. Here, we conducted a retrospective study to determine the impact on patient outcomes of the Xpert MTB/RIF test vs. phenotypical drug susceptibility testing for spinal TB.

**Methods:**

In-patients with spinal TB were enrolled in 2013 and 2017 at Beijing Chest Hospital. Data were collected from an electronic patient record system that documented demographic and clinical characteristics. All the patients were routinely followed-up at 1, 3, 6, 9, and 12 months after surgery during outpatient treatment.

**Results:**

A total of 361 patients affected by spinal TB were enrolled in our analysis, including 178 patients in 2013 and 183 patients in 2017. In 2013, the cumulative postoperative recurrence rate of patients with spinal TB was 23% (41/178), which was significantly higher than that in 2017 (8.2%, 15/183, *P* < 0.001). Additionally, the patients with spinal TB diagnosed in 2013 relapsed significantly sooner than those in 2017 (*P* < 0.001). In the multivariate analysis, rifampicin (RIF) resistance was associated with the recurrence of spinal TB. The turnaround time of Xpert ranged from 1 to 3 days, with a median of 1 day (IQR: 1–2). For the phenotypic drug susceptibility test (pDST)-based algorithm, the median turnaround time was 67 days, considerably longer than that of the Xpert-based algorithm (*P* < 0.001).

**Conclusion:**

The RIF resistance is an independent risk factor for postoperative recurrence in patients with spinal TB. Early detection of RIF resistance due to the application of Xpert is an effective strategy to reduce spinal TB recurrence.

## Introduction

Despite great efforts over the past decades, the global burden of tuberculosis (TB), caused by *Mycobacterium tuberculosis (*MTB*)*, remains substantial, with an estimated 10 million incident cases and 1.4 million deaths annually (1, 2). It primarily affects the lungs, but also affects other sites of the body, namely extrapulmonary TB (EPTB) (3, 4). According to the estimation by World Health Organization (WHO), EPTB represented 15% of the global TB burden (1). Dramatic increases in the incidence of EPTB have occurred in developed countries with low TB burden (1, 3, 5). Due to its paucibacillary nature and a wide variety of clinical manifestations, the diagnosis of EPTB is more difficult than pulmonary TB, which causes increased morbidity and lifelong disabilities (6). Thus, there is an urgent need for better diagnostic tools to accurately diagnose EPTB in resource-limited settings.

Spinal TB is one of the most common forms of EPTB, accounting for approximately two-thirds of all cases of skeletal TB (7). The progression of spinal TB is slow and insidious (8). The duration from symptoms to diagnosis varies from a few months to a few years, depending on accessibility to health care facilities (9). Surgical treatment plus antituberculosis therapies are the major medical procedures for the management of spinal TB (8). Although the surgical treatment does not different for drug-sensitive and drug-resistant TB, the chemotherapy regimens vary greatly in patients with different drug susceptibilities. As a consequence, rapid diagnosis of patients with spinal TB affected by drug-resistant tubercle bacilli is of paramount importance to improve clinical outcomes.

While phenotypic drug susceptibility is still regarded as the gold standard for detecting the susceptibility of MTB to various drugs, it has the limitation of a long turnaround time of several weeks due to the slow growth rate of tubercle bacilli. Additionally, the culture-based DST methods require a biosafety category 3 laboratory facility that is largely inaccessible in developing countries (10–12). The Xpert MTB/RIF assay (Cepheid, Sunnyvale, CA, USA), an automated real-time PCR system, is being widely implemented in decentralized settings, which increases the access to testing of EPTB specimens (13). In 2013, it is endorsed by WHO for simplification and fastening of EPTB diagnosis (14). More importantly, its potential to assess drug susceptibility accelerates the time to identify RIF-resistant TB. Given the low recovery rate of mycobacteria from various skeletal specimens (15), patients with spinal TB could benefit the most from this newer nucleic acid amplification test (NAAT) that results in early treatment initiation. Unfortunately, limited studies have assessed the effects of Xpert MTB/RIF on patients with TB, and most are from pulmonary TB (16, 17). In this study, we conducted a study to determine the impact of the Xpert MTB/RIF test on patient outcomes compared to phenotypic drug susceptibility testing for spinal TB in Beijing, China.

## Methods

### Study Design

We did a comparative study at the Beijing Chest Hospital in Beijing, China, between 2013 and 2017, to assess the impact of Xpert MTB/RIF on outcomes of adults hospitalized with spinal TB. We recruited all the definite in-patients with spinal TB, requiring surgery during the study period. This hospital was a tertiary referral TB hospital with about 900 beds. As the designated National Clinical Center on Tuberculosis, it provides health care for both patients with pulmonary TB and EPTB from Beijing Municipality and surrounding regions in Northern China (4). For patients with symptoms suggestive of spinal TB, such as fever, weight loss, night sweat, generalized body aches, and prominent spinal deformity, two skeletal specimens were obtained following biopsy of the lesion. One was used for laboratory examinations, including smear microscopy, mycobacterial culture, and/or Xpert MTB/RIF assay; the other was used to assess histological features indicative of tuberculosis. The rollout of Xpert MTB/RIF started in 2016 in our hospital. The patients with at least one component of the diagnostic criteria were diagnosed as definite spinal TB cases: i) smear-positive, culture-positive, and/or NAAT-positive specimen; ii) positive histology (i.e., the presence of acid-fast bacilli in Zeihl-Neelsen-stained histological section, and granulomatous inflammation with central necrosis in tissue lesion) (7).

### Laboratory Examinations

One direct smear was prepared per abscess specimen using routine auramine O fluorescent staining as previously described (18). Growth Indicator Tube (MGIT) was performed according to the standard method. Briefly, one milliliter of the abscess sample was decontaminated in N-acetyl-L-cysteine-NaOH-Na citrate (1.5% final concentration). After incubation for 15 min at room temperature, the digested sample was neutralized with phosphate buffer (PBS, 0.067 mol/L, pH = 7.4), followed by centrifugation at 4,000 g for 15 min, the sediment was resuspended in 2-ml PBS, and 0.5 ml of which was inoculated into an MGIT tube supplemented with oleic acid-albumin-dextrose-catalase (OADC) and antimicrobial supplement (polymyxin B, amphotericin B, nalidixic acid, trimethoprim, and azlocillin) (PANTA). Then the MGIT tube was incubated in the Bactec MGIT 960 system at 37°C. The growth of mycobacteria was automatically reported by the instrument (15). All positive cultures were identified by the MPT64 test as previously described (19).

For the Xpert assay, 1 ml of the abscess sample was mixed with 2 ml of sample reagent. After incubation for 15 min at room temperature, 2 ml of the inactivated sample reagent-sample mixture was transferred to the Xpert test cartridge. Cartridges were loaded into the GeneXpert device, and the results were automatically generated results after 120 min (20).

The positive cultures by MGIT were subcultured for phenotypic drug susceptibility testing (DST) purposes. The absolute concentration method was performed to determine the drug susceptibilities of MTB isolates for isoniazid (INH), rifampicin (RFP), ethambutol (EMB), and streptomycin (SM) (21), and the concentrations of drugs in media were as follows: RFP, 40 μg/ml; INH, 10 μg/ml; EMB, 0.2 μg/ml; and SM, 2 μg/ml.

### Data Collection

Data were collected from an electronic patient record system that documented demographic and clinical characteristics, including gender, age, ethnicity, place of residence, previous TB episode(s), comorbidities, treatment, and care over the in-patient period. In addition, the data on follow-up of patients were collected from the outpatient medical record system. Considering that the analysis data set did not include any patient identifiers, the study protocol was approved by the Ethics Committee of Beijing Chest Hospital under a waiver of informed consent.

### Outcomes

All the patients were routinely followed-up at 1, 3, 6, 9, and 12 months after surgery during outpatient treatment. The favorable outcome was defined as marked clinical improvement, whereas the poor outcome was defined as the re-emergence of located abscesses and unresolved clinical symptoms during the follow-up period. For the study cohort, the primary outcome was the frequency of RIF-resistant spinal TB; the second outcome was the proportion of patients experiencing postoperative recurrence.

### Statistical Analysis

Numbers and proportions of cases were tabulated according to multiple demographic and clinical factors associated with spinal tuberculosis. Chi-square analysis was performed to compare the clinical characteristics stratified by year. We used crude and adjusted Cox proportional hazard regression analysis to identify and evaluate factors impacting recurrence, including demographic characteristics, drug susceptibility profiles, and complications. All calculations in this study were conducted by using SPSS version 19.0 for Microsoft Windows (SPSS Inc., http://www.spss.com.hk). The difference was declared significant if the *P-value* was < 0.05.

## Results

### Patients

**Figure 1:**
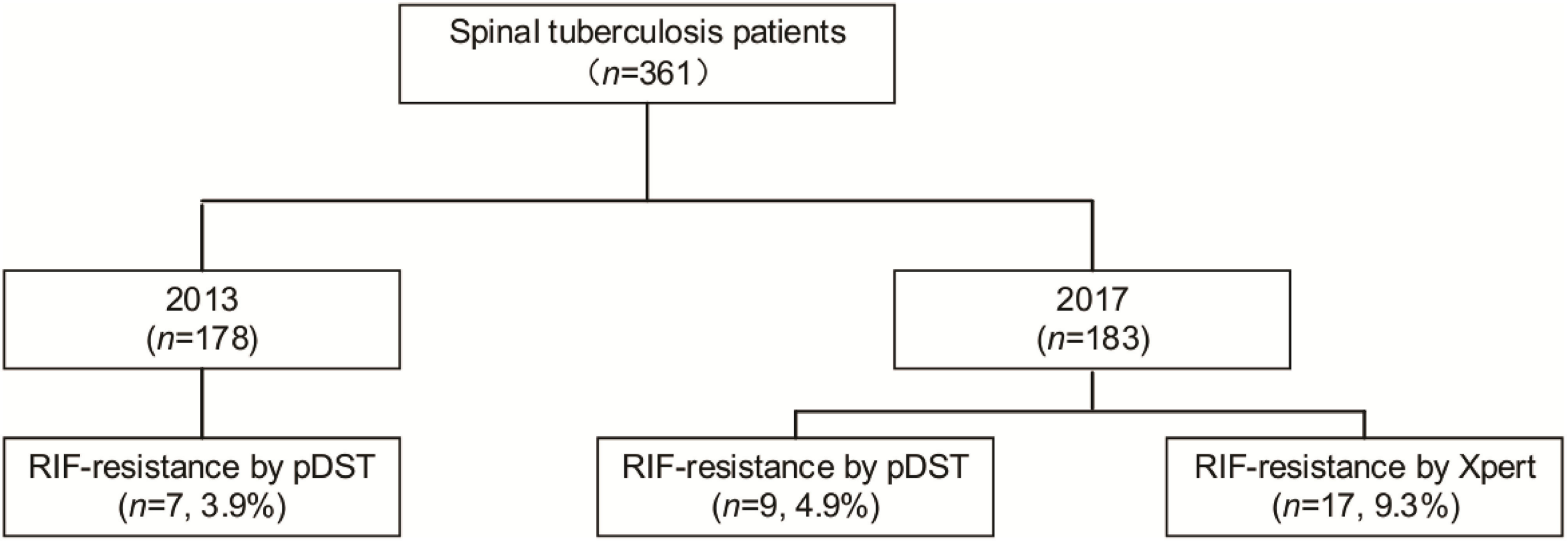
Comparison of patients with rifampicin (RIF)-resistant spinal tuberculosis between 2013 and 2017.

**Figure 2:**
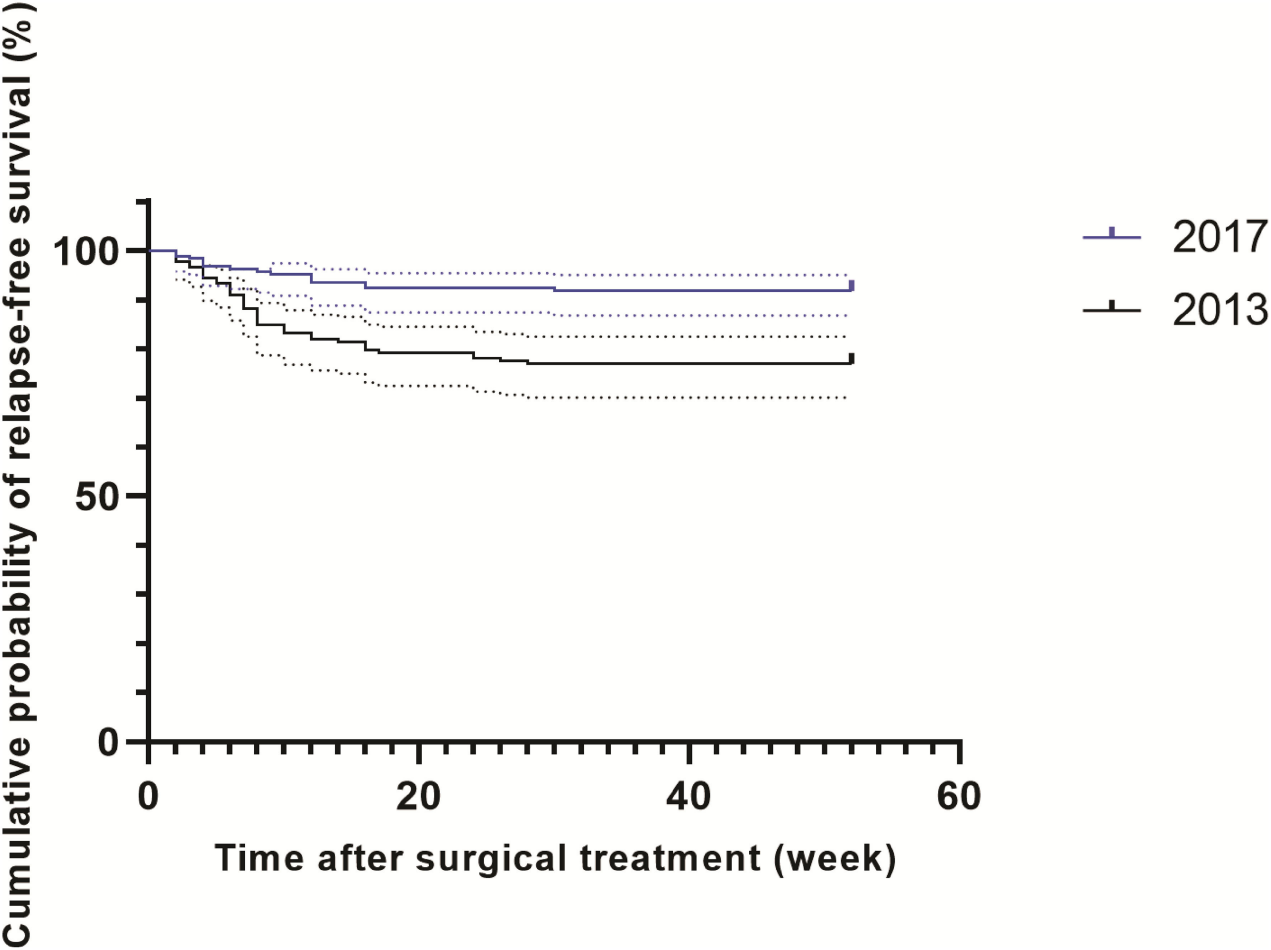
Cumulative probability of relapse-free survival in patients with spinal TB.

A total of 361 patients affected by spinal TB were enrolled in our analysis, including 178 patients in 2013 and 183 patients in 2017 (Figure 1). In 2013, of 178 patients, 7 (3.9%, 7/178) were identified as RIF-resistant by phenotypical drug susceptibility testing (pDST). Additionally, there were 9 (4.9%, 9/183) and 17 (9.3%, 17/183) patients diagnosed as RIF-resistant by pDST and Xpert in 2017, respectively. One hundred and eighty-four men with spinal TB were eligible in our cohort. The previous TB episode was identified in 81 patients (22.4, 81/361). The majority of patients with spinal TB were identified as lumbar TB, accounting for 50.7% of enrolled patients. A comparison in demographic and clinical characteristics of patients between 2013 and 2017 is summarized in Table 1. In 2013, the cumulative postoperative recurrence rate of patients with spinal TB was 23% (41/178), which was significantly higher than that in 2017 (8.2%, *P* < 0.001). In contrast, there were no other differences regarding demographic and comorbidities noted between 2013 and 2017. We further analyzed the cumulative probability of relapse-free survival between 2013 and 2017. As shown in Figure 2, the patients with spinal TB diagnosed in 2013 relapsed significantly sooner than those in 2017 (*P* < 0.001).

**Table 1:** Demographic and clinical characteristics of 2013 and 2017.

**Characteristics**	**2013**		**2017**		**Total**		**Chi–square**	***P***
	***n***	**%**	***n***	**%**	***n***	**%**		
Sex							1.794	0.180
Female	76	42.7	91	49.7	167	46.3		
Male	102	57.3	92	50.3	194	53.7		
Age groups, y							0.732	0.694
≤ 44	90	50.6	94	51.4	184	51.0		
45–59	52	29.2	47	25.7	99	27.4		
≥60	36	20.2	42	23.0	78	21.6		
Occupation							3.495	0.174
farmer	92	51.7	90	49.2	182	50.4		
staff member	42	23.6	33	18.0	75	20.8		
Others	44	24.7	60	32.8	104	28.8		
Residence							0.563	0.453
Rural	105	59.0	115	62.8	220	60.9		
Urban	73	41.0	68	37.2	141	39.1		
Treatment history							0.072	0.789
No	137	77.0	143	78.1	280	77.6		
Yes	41	23.0	40	21.9	81	22.4		
Classification							0.026	0.872
Thoracic TB	87	48.9	91	49.7	178	49.3		
Lumbar TB	91	51.1	92	50.3	183	50.7		
Abscess							0.738	0.390
No	50	28.1	59	32.2	109	30.2		
Yes	128	71.9	124	67.8	252	69.8		
Recurrence							15.156	<0.001
No	137	77	168	91.8	305	84.5		
Yes	41	23	15	8.2	56	15.5		
Diabetes							1.643	0.200
No	163	91.6	160	87.4	323	89.5		
Yes	15	8.4	23	12.6	38	10.5		
Immune diseases							<0.001	1.000
No	174	97.8	179	97.8	353	97.8		
Yes	4	2.2	4	2.2	8	2.2		
Culture determination								
Negative	134	75.3	126	68.9	260	72.0	1.851	0.174
Positive	44	24.7	57	31.1	101	28.0		
Drug sensitivity test							0.026	0.871
Sensitive	37	84.1	52	85.2	89	84.8		
Resistance	7	15.9	9	14.8	16	15.2		

### Factors Associated With Recurrence of Spinal Tuberculosis

We further investigated the factors associated with the recurrence of spinal TB in our study. In the univariate analysis, the presence of abscesses before surgical treatment and resistance were associated with recurrence of spinal TB; whereas only infection with RIF-resistant MTB was the sole risk factor contributing to recurrence [aHR: 4.682, 95%CI (1.712–12.808)]. However, the recurrence of spinal TB was not associated with sex, age group, diabetes, and immune dysfunction (Table 2).

**Table 2:** Risk factors for recurrence among Spinal Tuberculosis in the study.

**Characteristics**	**No recurrence**		**Recurrence**		**Total**		**Crude HR**	***P* value**	**Adjusted HR**	***P* value**
	***n***	**%**	***n***	**%**	***n***	**%**	**(95% CI)**		**(95% CI)**	
Sex										
Female	141	46.2	26	46.4	167	46.3				
Male	164	53.8	30	53.6	194	53.7	0.988 (0.584–1.670)	0.964		
Age groups, y										
≤ 44	156	51.1	28	50.0	184	51.0				
45–59	84	27.5	15	26.8	99	27.4	0.966 (0.516–1.808)	0.913		
≥60	65	21.3	13	23.2	78	21.6	1.094 (0.567–2.113)	0.788		
Treatment history										
New case	241	79.0	39	69.6	280	77.6				
Retreated case	64	21.0	17	30.4	81	22.4	1.538 (0.870–2.718)	0.139		
Classification										
Thoracic TB	153	50.2	25	44.6	178	49.3				
Lumbar TB	152	49.8	31	55.4	183	50.7	1.229 (0.726–2.082)	0.443		
Abscess										
No	99	32.5	10	17.9	109	30.2				
Yes	206	67.5	46	82.1	252	69.8	2.095 (1.057–4.152)	0.034	0.668 (0.182–2.444)	0.542
Drug sensitivity test										
Susceptible	79	89.8	10	58.8	89	84.8				
Resistant	9	10.2	7	41.2	16	15.2	4.346 (1.652–11.437)	0.003	4.682 (1.712–12.808)	0.003
Culture determination										
Negative	221	72.5	39	69.6	260	72.0				
Positive	84	27.5	17	30.4	101	28.0	1.152 (0.651–2.036)	0.627		
Diabetes										
No	271	88.9	52	92.9	323	89.5				
Yes	34	11.1	4	7.1	38	10.5	0.621 (0.225–1.717)	0.359		
Immune diseases										
No	299	98.0	54	96.4	353	97.8				
Yes	6	2.0	2	3.6	8	2.2	1.723 (0.42–7.069)	0.450		

*HR, Hazard Rate; CI, confidence interval*.

### Turnaround Time of Xpet and pDST-Based Algorithm

**Figure 3:**
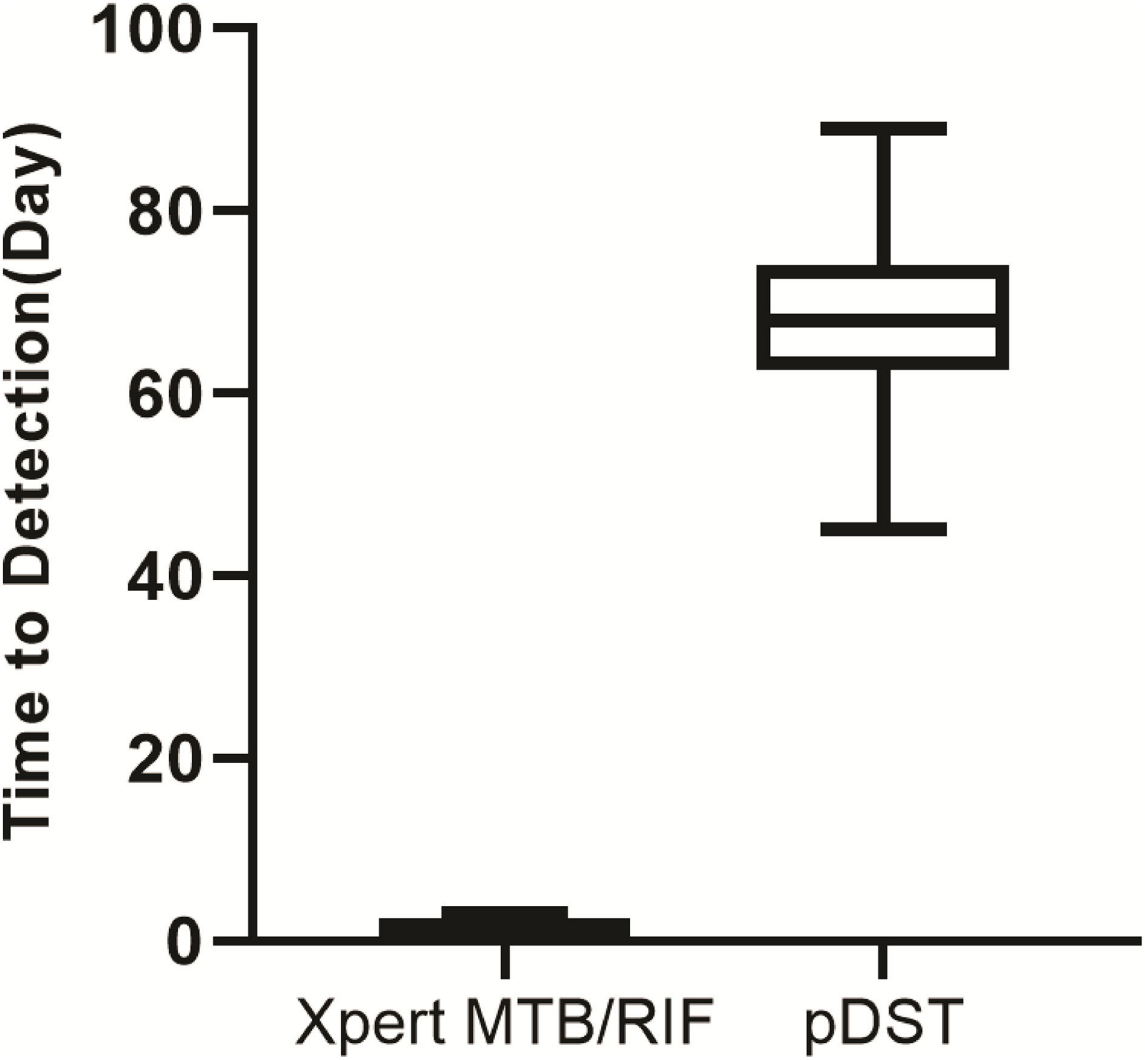
Comparative analysis of turnaround time between Xpert Myobacterium tuberculosis (MTB)/RIF and phenotypical drug susceptibility testing (DST) in 2017. pDST, phenotypical drug susceptibility testing.

The turnaround time in Xpert and pDST-based algorithms were compared in a routine operational setting. The turnaround time of Xpert ranged from 1 to 3, with a median of 1 day (IQR: 1–2). For the pDST-based algorithm, the median turnaround time was 67 days, which was considerably longer than that of the Xpert-based algorithm (*P* < 0.001) (Figure 3).

## Discussion

In the present study, our data demonstrated that the cumulative postoperative recurrence rate of patients with spinal TB was significantly decreased between the year before and the year after initiation of Xpert. Remarkably, approximately one-fourth of patients with spinal TB experienced postoperative recurrence in 2013. Similar results were reported by Wang and colleagues that recurrence was reported in 21 (24%) out of 87 patients with spinal tuberculosis (22). By contrast, in a retrospective study in Henan, the recurrence rate of bone TB was only 6% (23), which was significantly lower than our result. This difference may have been majorly explained by diversity in the inclusion of patients with various severity across studies. As the national clinical center on TB, our hospital accepts severe TB case referrals for patients originating from all regions of China; and the more severe condition at diagnosis undoubtedly leads to a higher recurrence rate. In addition, the length of the follow-up period was not stated in the latter study. The recurrence rate may be underestimated by loss of follow-up.

Previous studies have indicated that the infection with drug-resistant tubercle bacilli serves as a major driver of treatment failure in patients with TB (22, 24). In fair agreement with the earlier data, we found that RIF resistance is the sole independent risk factor for postoperative recurrence in multivariate analysis. Spinal tuberculosis remains a diagnostic dilemma due to difficulty in obtaining positive cultures, thereby preventing further determination of drug susceptibility. Therefore, the diagnosis delay of RIF resistance results in the empirical use of ineffective first-line regimens. The remaining drug-resistant tubercle bacilli, due to incomplete lesion removal, would multiply at a high rate, and lead to the onset and recurrence of spinal TB. Given the rich vascular supply of the vertebra (25), multiple vertebral bodies are simultaneously affected by tuberculosis, which increases the difficulty of surgery to completely remove the destroyed tuberculosis lesions. Our findings highlight that the early diagnosis of RIF resistance and initiation of appropriate treatment are essential to prevent the postoperative recurrence of spinal TB.

Of note, the recurrence rate is dramatically decreased from 23% in 2013 to 8.2% in 2017. This phenomenon could be majorly explained by the clinical application of the Xpert MTB/RIF assay. On one hand, an etiological diagnosis of spinal TB is not always obtained even when invasive techniques are used because abscess specimens contain masses of killed bacteria and dead neutrophils (26). Although the viability of pathogens significantly affects the recovery of tubercle bacilli, the Xpert assay does not require viable bacteria and is, therefore, less influenced by their viability (27). This hypothesis is confirmed by our observation that the yield of Xpert assay is almost twice that of pDST for detection of RIF resistance in 2017. On the other hand, performing Xpert as an initial test for spinal TB significantly decreases the turnaround time of results compared with phenotypic DST, thus, minimizing patient retention and generation of resistance. It is noteworthy that the majority of recurrent patients occurred within 12 weeks of follow-up, raising concerns as to whether the pDST could meet clinical criteria for the management of patients with drug resistance and with spinal TB. In our previous study, the rate of drug resistance in extrapulmonary patients with TB has significantly increased over the years (4). Taken together, our data highlight the use of Xpert as a preferable diagnostic for faster detection of RIF resistance, which is of great importance in reducing the postoperative recurrence of patients with spinal TB.

We also acknowledged several limitations to this study. First, this study only compared 1-year observational treatment results between the year before and year after initiation of Xpert in a single-center cohort. The small sample size may weaken the significance of our conclusion. Second, given the fact that no new antimicrobial agents have been warranted in skeletal patients since 2,000, the treatment regimens were not taken into consideration when analyzing the recurrence. However, the frequency of available anti-TB drugs in cohorts between 2 years may influence our analysis results. Finally, although the application of Xpert MTB/RIF could significantly decrease the incidence of postoperative recurrence, 8.2% of patients recurred in 2017, thus, suggesting the potential diagnostic failure of RIF resistance among these patients. Recently, the improved Xpert Ultra outperforms Xpert in diagnosing paucibacillary EPTB (28). Further studies are urgently needed to confirm the impact of this novel assay on the clinical management of patients with spinal TB.

To conclude, our findings demonstrate that RIF resistance is an independent risk factor for postoperative recurrence in patients with spinal TB. Early detection of RIF resistance due to the application of Xpert is an effective strategy to reduce their recurrence. Therefore, these data highlight the use of Xpert as a preferable diagnostic for faster detection of RIF resistance, which is of great importance in reducing the postoperative recurrence of patients with spinal TB.

## Data Availability Statement

The raw data supporting the conclusions of this article will be made available by the authors, without undue reservation.

## Ethics Statement

The study was approved by the Ethic Committee of Beijing Chest Hospital, Capital Medical University. Written informed consent for participation was not required for this study in accordance with the national legislation and the institutional requirements.

## Author Contributions

YP design of the work. JF, JA, and WS wrote the paper. JF, KT, JA, and SQ collected the data. YS and YX tested in laboratory. WS analyzed the data. All authors approved the final version to be submitted for consideration for publication.

## Funding

Beijing Hospitals Authority Ascent Plan (DFL20191601).

## Conflict of Interest

The authors declare that the research was conducted in the absence of any commercial or financial relationships that could be construed as a potential conflict of interest.

## Publisher's Note

All claims expressed in this article are solely those of the authors and do not necessarily represent those of their affiliated organizations, or those of the publisher, the editors and the reviewers. Any product that may be evaluated in this article, or claim that may be made by its manufacturer, is not guaranteed or endorsed by the publisher.
